# List of Diseases in London

**Published:** 1814-05

**Authors:** 


According to the report of several *Apothecaries* residing in various districts of the metropolis, the following was the average of the diseases in London between February 20th and March 19th:—

| | | | |
|---|---|---|---|
| Amentia | 1 | Cephalæa | 2 |
| Anasarca | 22 | Chlorosis | 17 |
| Angina Pectoris | 1 | Cholera | 18 |
| Ascites | 12 | Chorea | 1 |
| Asthma | 183 | Cephalalgia | 44 |
| Acne | 2 | Colica | 21 |
| Apoplexia | 19 | Catarrhus | 515 |
| Aphtha | 17 | Convulsio | 33 |
| Amenorrhœa | 30 | Carditis | 1 |
| Asthenia | 56 | Cynanche Tonsillaris | 105 |
| Abortio | 23 | ——— maligna | 8 |
| Bronchitis acuta | 24 | ——— Trachealis | 4 |
| ——— chronica | 26 | ——— parotidea | 20 |
| Cancer | 6 | Cystitis | 1 |

Diabetes

| | | | |
|---|---|---|---|
| Diabetes | 2 | Nausea | 1 |
| Dysenteria | 17 | Nephritis | 7 |
| Diarrhœa | 108 | Obstipatio | 63 |
| Dyspnœa | 55 | Ophthalmia | 60 |
| Dyspepsia | 153 | Palpitatio | 1 |
| Dysuria | 7 | Paralysis | 15 |
| Ecthyma | 8 | Phthisis Pulmonalis | 49 |
| Erythema | 5 | Pertussis | 92 |
| Erysipelas | 61 | Peritonitis | 14 |
| Enteritis | 12 | Phrenitis | 4 |
| Entrodynia | 19 | Pneumonia | 147 |
| Enuresis | 6 | Pleurodyne | 4 |
| Epilepsia | 12 | Podagra | 33 |
| Febris remittens | 133 | Porrigo larvalis | 6 |
| —— intermittens | 22 | —— scutulata | 5 |
| —— puerpera | 5 | —— favosa | 4 |
| Fungus Hæmatodes | 1 | Psoriasis | 13 |
| Gastritis | 2 | Purpura | 2 |
| Gastrodynia | 45 | Pyrosis | 10 |
| Hæmatemesis | 2 | Rachitis | 3 |
| Hæmaturia | 2 | Rheumatismus acutus | 92 |
| Hæmoptoe | 27 | —— chronicus | 105 |
| Hæmorrhois | 33 | Rubeola | 22 |
| Hepatitis | 33 | Scabies | 95 |
| Hysteria | 34 | Scarlatina simplex | 20 |
| Hysteritis | 3 | —— anginosa | 14 |
| Hydrocephalus | 22 | —— maligna. | 1 |
| Hydrothorax | 10 | Splenitis | 3 |
| Herpes | 28 | Scrofula | 30 |
| Hypochondriasis | 15 | Synochus | 8 |
| Icterus | 16 | Tabes Mesenterica | 12 |
| Impetigo | 13 | Tic Douloureux | 1 |
| Ischuria | 13 | Trismus | 1 |
| Leucorrhœa | 36 | Typhus | 10 |
| Lithiathis | 1 | Variola | 4 |
| Lepra | 2 | Varicella | 2 |
| Lichen | 2 | Vermes | 33 |
| Mania | 13 | Vertigo | 26 |
| Menorrhagia | 30 | Urticaria | 10 |
| Miliaria | 1 | | |
| Morbi Infantiles | 197 | Total | 3623 |
| —— Biliosi | 172 | | |

The Deaths in the Bills of Mortality from February 15th to March 15th, 1814, according to the returns of the *Parish Clerks*, were as under:—

| | |
|---|---|
| Males . . . . | 913 |
| Females . . . | 778 |
| Total | 1691 |

LONDON

